# Frugal inference for control

**Published:** 2025-09-03

**Authors:** Itzel Olivos-Castillo, Paul Schrater, Xaq Pitkow

**Affiliations:** 1 Department of Computer Science, Rice University.; 2 Department of Computer Science, University of Minnesota.; 3 Department of Psychology, University of Minnesota.; 4 Neuroscience Institute, Carnegie Mellon University.; 5 Department of Machine Learning, Carnegie Mellon University.; 6 Department of Neuroscience, Baylor College of Medicine.; 7 Department of Electrical and Computer Engineering, Rice University.; 8 Center for Neuroscience and Artificial Intelligence, Baylor College of Medicine.; 9 NSF AI Institute for Artificial and Natural Intelligence.

## Abstract

A key challenge in advancing artificial intelligence is achieving the right balance between utility maximization and resource use by both external movement and internal computation. While this trade-off has been studied in fully observable settings, our understanding of resource efficiency in partially observable environments remains limited. Motivated by this challenge, we develop a version of the POMDP framework where the information gained through inference is treated as a resource that must be optimized alongside task performance and motion effort. By solving this problem in environments described by linear-Gaussian dynamics, we uncover fundamental principles of resource efficiency. Our study reveals a phase transition in the inference, switching from a Bayes-optimal approach to one that strategically leaves some uncertainty unresolved. This frugal behavior gives rise to a structured family of equally effective strategies, facilitating adaptation to later objectives and constraints overlooked during the original optimization. We illustrate the applicability of our framework and the generality of the principles we derived using two nonlinear tasks. Overall, this work provides a foundation for a new type of rational computation that both brains and machines could use for effective but resource-efficient control under uncertainty.

## 1 Introduction

Smart actions should consider the long-term consequences of perceptual and movement errors. Such errors are inevitable in natural environments where relevant variables are hidden and constantly changing. A well-known and powerful framework for modeling how this uncertainty evolves and impacts long-term outcomes is the Partially Observable Markov Decision Process (POMDP). This framework allows reliable decision-making by supporting interpretable reasoning, principled exploration, and generalization grounded in posterior distributions that extend beyond specific histories; however, identifying the sequence of actions that maximizes expected utility in a POMDP is challenging [1, 2]. A central difficulty lies in the need to construct and continually update a posterior distribution over the hidden variables, an inference process that is rarely tractable.

Sampling-based methods [3, 4] and variational techniques [5–9] are common, tractable strategies for approximate inference. In principle, these approaches can yield high-quality approximations of the Bayes-optimal posterior, the “belief” that summarizes the history of past observations and actions. However, achieving this level of accuracy can still be computationally demanding, requiring the simulation of large particle sets or the optimization of highly expressive variational families. This computational burden often overwhelms the capacity of real-world agents. For example, NASA rovers operate with radiation-hardened microchips that prioritize reliability over processing speed and must judiciously allocate limited energy across sensing, inference, control, communication, and thermal regulation. Similarly, robots such as autonomous vacuums, lawnmowers, and pool cleaners must minimize hardware costs to remain commercially viable, which typically rules out using specialized parallel computing hardware.

When operating under realistic resource constraints such as time, memory, energy, and computing power, effective performance hinges on the agent’s ability to optimize the trade-off between resource use and utility improvements. This trade-off was first recognized in Simon’s pioneering work on bounded rationality [10] and later formalized in models such as bounded optimality [11] and meta-reasoning [12–14]. Building on these foundations, information-theoretic frameworks [15–18] have advanced resource-efficient planning in fully observable domains. These frameworks connect the normative structure of decision theory with the quantitative limits of information processing, providing theoretical insights that are agnostic of hardware specifics and implementation details. This line of research has produced scalable implementations [19], and been extended to settings with model uncertainty [20]. It has gained empirical support from studies showing that information-theoretic constraints can account for the emergence of heuristics in human decision making [21], explain how people balance reward maximization with cognitive effort [22], and characterize the pervasive coexistence of habitual and controlled responses across many tasks [23]. Despite this progress, our understanding of resource efficiency in partially observable environments remains limited. Most studies focus on explaining cognitive-level behavior without pursuing the computational implementations [24–26]. Other works showcase practical implementations in complex tasks but do not seek to derive theoretical insights [27–29]. The few works that both aim to derive principles and demonstrate applicability are restricted to single-step decision-making problems [30–33] or settings that solely address perception or communication constraints [34–36]. To our knowledge, understanding how the computational burden of inference affects rational behavior in complex, partially observed control tasks remains an open problem, explored primarily at a conceptual level [14, 37, 38].

Motivated by this challenge, we formulate the computational burden of inference in information-theoretic terms and propose a variant of the POMDP framework in which inference is treated as a regulated process rather than a fixed subroutine. This introduces a new trade-off to the usual one between achieving the goals and the effort to get there: we now add a trade-off of those aspects of task performance against the cost of representing information distilled from previous evidence. To understand the trade-offs among these competing objectives, we solve a simple version of the general problem, focusing on the special case of linear dynamics with Gaussian variability, and thoroughly characterize the solutions. Our study reveals that complex properties emerge even in this simple setting: we observe a phase transition in the inference, switching from a Bayes-optimal approach to one that strategically leaves some uncertainty unresolved. This frugal behavior reshapes the optimization landscape of the planning problem. When the agent discards using Bayes-optimal inference, the solution comprises a family of strategies that differ in how the agent integrates new evidence and compensates for estimation errors. A free orthogonal transformation relates the family members; this additional freedom helps satisfy additional objectives or constraints that were overlooked during the original optimization. Using two nonlinear tasks, we illustrate the applicability of our framework and the generality of the principles derived from studying the linear-Gaussian setting. Overall, our findings extend the information-theoretic perspective on resource efficiency from fully observed to partially observed domains, generalize the principle of minimal intervention in control, and provide a foundation for a new type of rational computation that both brains and machines could use for effective but resource-efficient control under uncertainty.

## 2 Results

### 2.1 Control when information is costly

**Figure 1:**
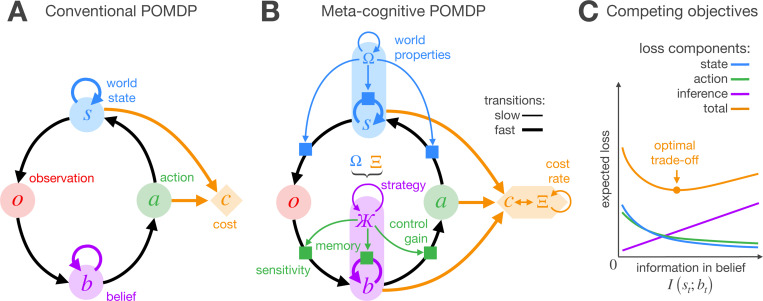
Structure of computationally constrained control. **A)** Conventional POMDP. The agent interacts with a hidden world state over time, receiving noisy observations, taking actions that change the state, and incurring costs based on the action taken and the resulting next state. Minimizing cumulative costs requires managing state uncertainty. To address this, the agent builds and updates a belief over the hidden state that aims to fully summarize previous evidence (the history of past observations and actions). **B)** Meta-cognitive POMDP. The agent pays for the information that beliefs encode about hidden world states. To balance this internal cost against state and action costs, the agent computes a strategy Ж that dictates how to integrate new evidence and how to transform the resulting beliefs into actions. To compute this strategy, the agent considers the properties of the world, Ω, and the penalty parameters of the loss function, Ξ; factors that we assume are fully observable and change slowly. **C)** Optimal trade-off. State and action costs decrease as the belief encodes more information about the hidden state. However, when information is costly, the agent can achieve greater utility by tolerating more state and action costs if doing so saves enough bits in the inference. This research highlights principles that allow optimizing this trade-off in POMDPs with linear-Gaussian dynamics.

In a conventional POMDP (Figure 1A), the set of task-relevant variables (world state $s_{t}$) is hidden. To mitigate this uncertainty, the agent uses probabilistic inference to build and update a belief $b_{t}$ that summarizes the history of previous observations $o_{\leq t}$ and actions $a_{<t}$. The more information the belief encodes about the hidden state, the more purposeful and effective the agent’s actions can be. However, every bit of information gained through inference comes at the cost of time, computation, memory, and energy. To investigate how accounting for this burden influences rational behavior, we develop a version of the POMDP framework where belief updating is considered an internal “cognitive” process that the agent can monitor and meta-regulate. Planning in the resulting meta-cognitive POMDP involves jointly optimizing inference and control (action selection) to minimize the following loss function:

(1) $$E\left[\sum_{t}s_{t}^{⊤}C_{s}s_{t}+a_{t}^{⊤}C_{a}a_{t}+C_{n}I\left(s_{t};b_{t}\right)\right]$$

where the penalties $\text{Ξ}=\left\{C_{s},C_{a},C_{n}\right\}$ encourage the agent to mitigate deviations from target states, $s_{t}^{⊤}C_{s}s_{t}$, reduce motion effort, $a_{t}^{⊤}C_{a}a_{t}$, and decrease the mutual information between states and beliefs, $C_{n}I\left(s_{t};b_{t}\right)$. To balance these competing objectives (Figure 1C), we assume the agent has exact knowledge of the penalty parameters, Ξ, and the world properties, Ω, that determine state transitions and observation generation. We further assume that Ξ and Ω change slowly over time, enabling the agent to gradually adapt its parameters for extended periods of stable control at equilibrium. During the adaptation phase, the agent computes a strategy $Ж=\left\{\phi^{b},\phi^{a}\right\}$ (using the cyrillic letter ‘zhe’) that dictates how to integrate previous evidence (inference process, parameterized by $\phi^{b}$) and how to transform the resulting beliefs into actions (control policy, parameterized by $\phi^{a}$). Following adaptation, the agent enters a long period of stable control, interacting with the world using the beliefs and actions derived from the strategy Ж, that is, $b_{t}=f\left(o_{\leq t},a_{<t};\phi^{b}\right)$ and $a_{t}=g\left(b_{t};\phi^{a}\right)$.

### 2.2 An interpretable testbed

In general settings, the parameters defining the strategy that optimizes the trade-off among state, action, and inference costs can be complex, such as the weights of a recurrent neural network. However, the solution is tractable and more interpretable for linear-Gaussian POMDPs. In this setting, the hidden state evolves according to stochastic linear dynamics, $s_{t}=Ds_{t-1}+Ea_{t-1}+w_{t-1}$, and observations are linear, noisy versions of the hidden state $o_{t}=s_{t}+v_{t}$. Here, the dynamics matrix D captures how unstable the state is, the input gain E characterizes actuator responsiveness, $a_{t-1}$ is the action taken by the agent, $w_{t-1}$ is additive white Gaussian process noise with isotropic covariance Q, and $v_{t}$ is additive white Gaussian observation noise with isotropic covariance R.

Without our added internal inference cost, which penalizes the mutual information between hidden states and beliefs, this structure allows for an analytical solution—the LQG controller—which combines Bayes-optimal inference via a Kalman filter with optimal control via a linear quadratic regulator [39]. Due to this advantage, linear-Gaussian POMDPs are widely used as testbeds for uncovering fundamental principles in control theory [40], reinforcement learning [41], and neuroscience [36, 42, 43]. Beyond such foundational studies, the tractability of linear-Gaussian dynamics has also been leveraged to address complex, nonlinear, continuous-state control problems that would otherwise be intractable [44]. We, too, capitalize on the analytical tractability of this problem setting, but our solution does not rely on the LQG controller. Section 4 presents our method to solve meta-cognitive POMDPs with linear-Gaussian dynamics. We solve the planning problem at equilibrium, where the expected total cost is entirely defined by a steady-state covariance matrix Σ that captures the dependencies among states, observations, beliefs, and actions. The solution is an interpretable strategy: $\{\text{Π},\text{Ψ}\}=\left\{L\text{Γ}L^{+},L\beta \right\}$. Here, Γ and β parameterize the inference process: Γ defines how much of the past should be remembered and β determines how to scale observations to minimize estimation bias. The control gain L parameterizes the control policy by dictating how to translate the resulting beliefs into actions, and the symbol ^+^ denotes the Moore–Penrose pseudoinverse.

### 2.3 Principles of frugal inference in linear-Gaussian POMDPs

#### 2.3.1 Spend when it counts

**Figure 2:**
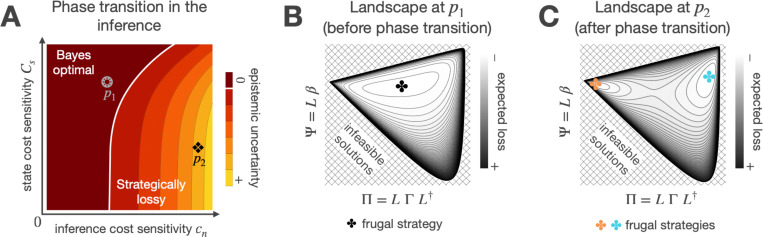
Parameter space for frugal inference. **A)** Phase transition in the optimal inference strategy. The penalties $C_{s}$ and $C_{n}$, which determine the relative importance of minimizing state deviations and reducing information use, set a threshold (white line) beyond which the benefits of Bayes-optimal inference saturate. Markers $p_{1}$ and $p_{2}$ indicate parameters at which the optimization landscapes of Plots B and C are defined. **B)** Optimization landscape before the phase transition. The optimization landscape of the planning problem is convex when the agent relies on Bayes-optimal inference. **C)** Optimization landscape after the phase transition. When the agent leaves some epistemic uncertainty unresolved, the optimization landscape has multiple global minima. The multiple solutions achieve statistically equivalent performance but differ in how the agent integrates new evidence, offsets estimation errors, and generalizes to novel settings.

Unburdened by any computational constraints, the optimal solution to control under uncertainty involves selecting actions based on beliefs derived from an inference process that mitigates reducible (epistemic) uncertainty and accurately quantifies irreducible (aleatoric) uncertainty. However, for agents operating under computational limitations, the demands of Bayes-optimal inference may exceed processing capacity. In such cases, conserving computational resources—by reducing the information distilled from previous evidence—emerges as an additional objective that competes against optimizing task performance (state cost) and motion effort (action cost). The solution to this computationally constrained control problem is a frugal strategy that aligns resource usage with task demand and environmental reliability. Figure 2A illustrates this behavior in a scalar task. The agent only invests in Bayes-optimal inference when it is affordable—meaning the cost per bit of information gained through inference, $C_{n}$, is low—or essential, which occurs when the cost per unit of deviation from the target state, $C_{s}$, is high. Otherwise, the agent relies on a lossy inference approach that strategically leaves some epistemic uncertainty unresolved. This phase transition in the inference, from Bayes-optimal to lossy, coincides with a shift in the optimization landscape of the planning problem from convex (Figure 2B) to having multiple global minima (Figure 2C). In the following section, we delve into the characteristics of the multiple solutions that emerge when the benefits of Bayes-optimal inference saturate.

#### 2.3.2 Adaptability begins when perfection ends

**Figure 3:**
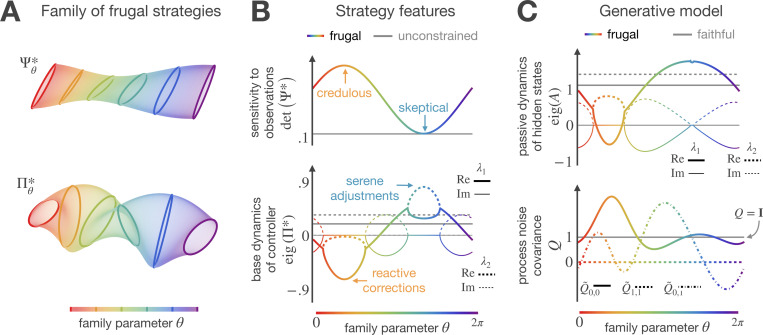
Family of frugal strategies. **A)** Graphical representation of optimized strategies. For 2-dimensional control tasks, the solutions are described by 2×2 matrices of observation sensitivity Ψ and controller’s base dynamics Π. Here these matrices are visualized by how they transform a unit circle into an ellipse. Members of this family of strategies are related by an orthogonal transformation that is fully defined by a free angle θ (depicted by hue). For each color there is a pair of ellipses for the surfaces of Ψ and Π, representing a strategic combination of lossy inference and error-aware control. **B)** Strategy features. The family members differ in how the agent integrates new evidence and offsets estimation errors. For instance, the strategies prioritizing observations over predictions require controllers that frequently change the direction of motion. In contrast, the strategies that prioritize predictions over observations rely on controllers that correct deviations with gradual, smooth movements. The combination of inference and control that solves the unconstrained control problem is shown in gray. **C)** Generative model. To save bits in the inference, the agent makes deliberately mistaken assumptions about the world. Some strategies model the stochasticity in the transition dynamics as stable oscillations with high process noise (orange-hued members); others explain this randomness as low process noise in a volatile environment (blue-hued members). The ground truth properties are shown in gray.

When the agent discards Bayes-optimal inference, the solution to the computationally constrained control problem becomes a family of frugal strategies. Intuitively, this occurs because, while there is only one way to achieve Bayes-optimality, there are many ways to make mistakes that leave some epistemic uncertainty unresolved. In Section 4.4, we provide the mathematical justification of this intuition. We also demonstrate that the solution family has structure: its members are related by a free orthogonal transformation, which allows the recovery of the entire family from a single solution. This transformation manifests as a reflection for scalar tasks, which accounts for the two global minima observed in Figure 2C. However, in the multivariate context, the transformation gives rise to countless combinations of perception and mobility. To demonstrate this, Figure 3A visualizes characteristics of a family solving a 2-dimensional task.

All family members yield statistically equivalent state, action, and internal inference costs; however, due to differences in their temporal structure, they differ in how the agent integrates new evidence and offsets estimation errors. The controller’s input-output form, $a_{t}=\text{Π}a_{t-1}+\text{Ψ}o_{t}$, allow us to explore these differences. Here, the controller’s base dynamics Π defines how the actions evolve without new evidence, while the observation sensitivity Ψ determines which dimensions can be attenuated or magnified without compromising eventual goal attainment. As Figure 3B illustrates, the solutions allow multiple distinct combinations of lossy inference and error-aware control. For example, the orange-hued strategies combine “credulous inference” with “reactive control,” which means they give new observations more credence compared to predictions, so they continuously reverse the direction of motion to reactively correct estimation errors. In contrast, the blue-hued solutions pair “skeptical inference” with “serene control,” tending to disbelieve observations and instead favor prior predictions, and they rely on a gradual correction of major deviations. The different ways family members integrate new evidence can be understood as Bayes-optimal inference based on a mistaken world model (Figure 3C). For example, the credulous and reactive strategy models the stochasticity in the world as stable oscillations coupled with high process noise. Conversely, the skeptical and serene solution interprets stochasticity as low process noise in a volatile environment.

Access to a structured family of diverse but equally effective strategies facilitates adaptation to new tasks. The orthogonal transformations between family members span a free design subspace that the agent can explore to satisfy additional objectives or constraints not initially considered during optimization, but without reducing performance on the original task.

#### 2.3.3 Thinking less, moving more

**Figure 4:**
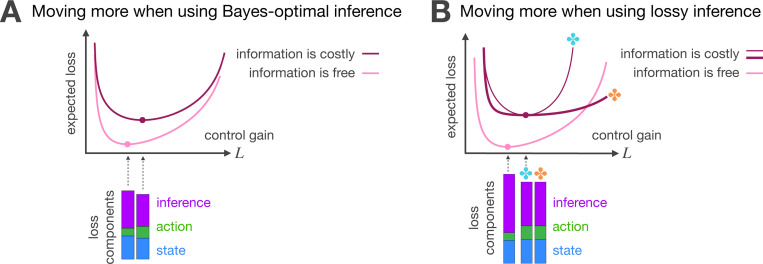
Trade-off between motion effort and inference cost. The best control gain with costly information is higher than the best control gain when information is free. This additional motion effort serves two purposes, depending on inference quality, as shown here: **A)** Additional motion when using Bayes-optimal inference. The agent applies a strong control gain to decrease state variance; this approach indirectly lowers inference cost by reducing the variability that previous evidence has to explain. **B)** Additional motion when using lossy inference. The agent applies a strong control gain to offset the estimation errors arising from unresolved uncertainty.

When information is costly $\left(C_{n}>0\right)$, agents trade motion effort for savings in the inference. This additional effort serves two purposes. On the one hand, when Bayes-optimal inference is needed to avoid catastrophic estimation errors (Figure 4A), the agent applies a stronger control gain to reduce state variance. This effort decreases the variability that previous evidence has to explain, indirectly lowering the information that can be extracted from previous evidence. On the other hand, when the agent chooses to leave some uncertainty unresolved (Figure 4B), a stronger control gain helps counteract the estimation errors that arise from cheaper inference.

### 2.4 Empirical validation in nonlinear environments

We selected two nonlinear tasks to illustrate the applicability of our framework and the generality of the principles derived from studying linear-Gaussian settings. In both cases, the agents are simplified models of practical machines operating under tight computational budgets. To study these systems, we first linearize the dynamics around the target state. Next, we compute the frugal strategies using the method outlined in Section 4. Finally, we assess performance by executing the strategies on the original nonlinear system.

#### 2.4.1 Meta-cognitive cart pole

**Figure 5:**
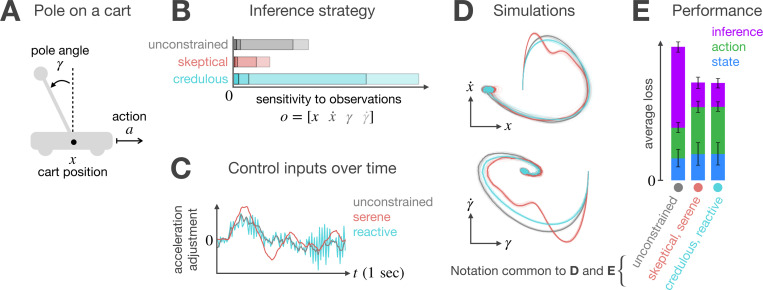
Frugal control for balancing a pole. **A)** Schematic of relevant variables. The controller aims to balance the pole on a moving cart by adjusting the cart’s acceleration. **B)** Inference sensitivity to observations. The unconstrained agent (gray) weighs observations based solely on statistical reliability. In contrast, frugal agents (non-gray) also take control objectives into account. This entails a strategic adjustment of observation weights that, when paired with a suitable control policy, optimizes information usage while still ensuring eventual goal attainment. **C)** Control trajectories for different agents. Skeptical inference can be compensated by a serene controller that adjusts the cart’s acceleration gradually. However, credulous inference requires a reactive controller that frenetically changes the direction of motion. **D)** State-space trajectories. Both frugal agents (non-gray trajectories) are able to attain the goal, stabilizing the pole at the upright position. Individual trials are displayed in light colors, with the mean trajectory emphasized in dark. **E)** Statistical performance at equilibrium. Although the frugal strategies differ noticeably during the transient, they incur statistically identical state, action, and inference costs at equilibrium. Error bars indicate one standard deviation

Our first task is a classic control problem: balancing a pole on a moving cart (Figure 5A). To balance the pole, the cart moves forward and backward. The action space is one-dimensional, with actions controlling the cart’s acceleration. The hidden state is a four-dimensional vector $\left(x,\dot{x},\gamma ,\dot{\gamma }\right)$, representing the cart’s position, velocity, pole angle, and angular velocity. Instead of observing the true state directly, the agent receives a four-dimensional noisy observation vector. Noise is present in all state variables, though it is more pronounced in the velocity and the angular velocity. A real-world application of this problem is the Segway—a self-balancing transportation device that allows tourists to explore cities while avoiding traffic jams. Devices like the Segway must carefully manage their energy consumption to remain operational for extended periods, making them an interesting case study for our meta-cognitive POMDP framework.

For an agent operating in a one-dimensional action space and prioritizing saving bits in the inference (high $C_{n}$), the solution to the planning problem is a family of two frugal strategies. We examine the behavior of agents implementing those strategies and compare their performance to that of an unconstrained agent $\left(C_{n}=0\right)$. When integrating new evidence (Figure 5B), the unconstrained agent adopts a purely statistical approach, weighting each dimension of the observation in proportion to its reliability. Frugal agents, in contrast, take into account both measurement reliability and control objectives. This allows saving bits in the inference by strategically adjusting observation weights and control gain without compromising eventual goal attainment. Figure 5C illustrates the two possible mechanisms that help mitigate the estimation errors arising from cheaper inference. Errors that result from attenuating observation weights (skeptical inference) are counteracted by smooth, gradual adjustments to the cart’s acceleration (serene control). However, offsetting the errors caused by amplifying observation weights (credulous inference) requires frenetic adjustments to the direction of motion (reactive control). Figure 5D illustrates the state-space trajectories that each strategy induces. Although the frugal strategies differ noticeably during the transient, both successfully drive the system to an equilibrium near the target state, keeping the pole upright. Statistical analysis of performance at equilibrium (Figure 5E) confirms that both frugal strategies solve the computationally constrained control problem equally well, achieving statistically equivalent state, action, and inference costs. While this equivalence may seem counterintuitive, given that one strategy produces smooth actions and the other exhibits frenetic fluctuations, the action cost remains identical because the loss function penalizes total control magnitude, not temporal variability. Thus, despite differing trajectories, the cumulative action cost is equivalent under our evaluation metric (Equation 1).

#### 2.4.2 Meta-cognitive drone

**Figure 6:**
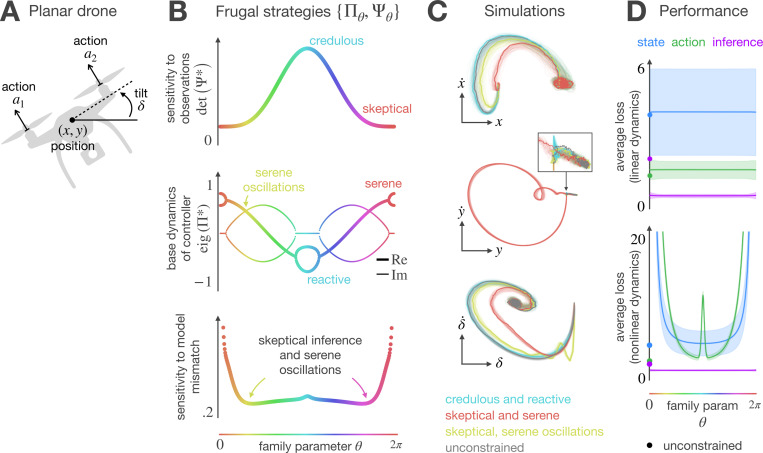
Frugal control of a planar drone maintaining a fixed hover position. **A)** Schematic of relevant variables. **B)** Family of frugal strategies. For a two-dimensional controller, the solution to the planning problem comprises infinite combinations of lossy inference and error-aware control. These strategies differ in how the agent integrates new evidence (top), offsets estimation errors (middle), and generalizes to novel settings (bottom). **C)** State-space trajectories. The frugal strategies successfully drive the system to an equilibrium near the target state. During the transient, combining skeptical inference with serene control yields state-space trajectories that differ substantially from those generated by an unconstrained agent, a credulous and reactive agent, and a serene agent with oscillations. This behavior is in line with our sensitivity analysis (panel B, bottom), which indicates that combining skeptical inference with serene control is highly sensitive to model mismatch. Here, the mismatch arises because the trajectories reflect the true nonlinear dynamics, whereas the strategies were computed using linearized approximations of those dynamics. Individual trials are displayed in light colors, with the mean trajectory emphasized in dark. **D)** Statistical performance at equilibrium. All family members perform equally well under linear dynamics (top), but respond differently when evaluated using simulations of the nonlinear model (bottom). Mean cost is shown by lines, with shaded regions denoting one standard deviation; unconstrained performance is marked by a dot on the vertical axis.

In our final set of experiments, we assess a drone’s ability to maintain a stable hover in the presence of gravity and external disturbances such as wind gusts. The drone is restricted to move within the xy-plane and is modeled as a rigid body equipped with two propellers (Figure 6A). The action space is two-dimensional: thrust commands to the propellers that jointly control the drone’s altitude and orientation. The hidden state is a six-dimensional vector $\left(x,y,\delta ,\dot{x},\dot{y},\dot{\delta }\right)$, representing the drone’s horizontal and vertical positions, tilt angle, and their respective velocities. The agent receives a six-dimensional observation vector subject to noise in all state variables, with the greatest impact on velocity components. Autonomous drones operate under severe resource limitations. Their batteries must support locomotion, communication, sensing, and planning, making these devices another interesting case study for our meta-cognitive POMDP framework.

For an agent operating in a two-dimensional action space while saving bits in the inference (high $C_{n}$), the solution to the planning problem is an infinite set of frugal strategies (Figure 6B). Although these multiple combinations of lossy inference and error-aware control achieve statistically equivalent performance under the linear model for which they were optimized, they respond differently to model perturbations. To examine their sensitivity to model mismatch, we introduce subtle variations in the drone’s mass and arm length—parameters that directly influence the motor responsiveness. We quantify robustness by computing the natural gradient of the expected loss with respect to these parameters, capturing the worst-case local sensitivity. As shown in Figure 6B-bottom, the frugal strategies differ markedly in their robustness. Notably, combining skeptical inference with serene control is very sensitive to model mismatch; however, a slight modification that introduces oscillations in the controller’s base dynamics generalizes best across all family members.

Figure 5C compares the state-space trajectories generated by three strategies in the solution family: ${Ж}_{1}$, which combines credulous inference and reactive control, ${Ж}_{2}$, which pairs skeptical inference with serene control, and ${Ж}_{3}$, which combines skeptical inference and serenely oscillating control. As expected, the frugal strategies generate noticeably different trajectories during the transient. Strategy ${Ж}_{1}$, which relies on frenetic corrections to estimation errors, induces trajectories that closely resemble those of an unconstrained agent $\left(C_{n}=0\right)$. The resemblance is convenient, but the frenetic corrections may increase actuator wear. Strategy ${Ж}_{2}$, which offsets estimation errors through smooth, gradual adjustments, is better suited for reducing actuator wear. However, smooth control comes at the expense of larger deviations from unconstrained performance. Interestingly, strategy ${Ж}_{3}$ strikes a balance between the two: it achieves state trajectories that resemble those of an unconstrained agent while relying on smooth, oscillatory actions that may reduce stress on hardware during implementation. These results show how the free design subspace arising when the agent discards Bayes-optimal inference can be leveraged to meet extra goals that were not relevant during the original optimization. Finally, Figure 5D presents the performance at equilibrium of the complete solution family. As expected, all family members perform equally well when evaluated using simulations of the linearized dynamics; however, under simulations of the nonlinear model, the strategies that combine skeptical inference and serene control exhibit poor generalization.

## 3 Discussion

We introduced a variant of the POMDP framework in which the information distilled from previous evidence is a resource the agent can meta-regulate. This creates a trade-off: an agent can obtain more utility overall by tolerating more state and action costs if doing so saves enough bits in the inference. In the multivariate context, this trade-off expands the principle of minimal intervention in control, which states that control should be exerted only on deviations that worsen the objective. Here, instead of just minimizing action costs, our agents also reduce inference costs by choosing which dimensions can be attenuated or magnified without compromising eventual goal attainment. By solving meta-cognitive POMDPs with linear-Gaussian dynamics and studying the solutions, we derived fundamental principles of resource efficiency. Our study reveals that a meta-cognitive agent only engages in Bayes-optimal inference when necessary or affordable; otherwise, it leaves some epistemic uncertainty unresolved and uses locomotion to counteract estimation errors. This frugal behavior reshapes the optimization landscape from convex to one with multiple global minima. The solutions that emerge when the agent chooses to leave some uncertainty unresolved form a structured family of equally effective strategies, a feature that facilitates adaptation to new tasks. Using two control problems involving the stabilization of nonlinear systems, we illustrated the applicability of our framework and the generality of the principles we derived.

While these results advance our understanding of how the computational burden of inference affects rational behavior in complex, partially observed control tasks, we acknowledge several important caveats. This study focused on simplified control problems, specifically LQG problems, which have historically been instrumental in revealing fundamental structures in computations [36, 40–43, 45]. Here, too, this simplification allowed us to thoroughly understand key new control principles that emerge with the introduction of computational constraints. Although such groundwork is essential, it also highlights important limitations that future work should address. We assumed a jointly gaussian system for process noise and observation noise, such that all covariances were independent of the controls, world states [42], and inference states; nor did we include internal computational noise [43]. Many real-world control problems involve much higher-dimensionality that we examined, like dynamic image processing in robotics for which computational demands are especially high. Finally, real control problems have nonlinear dynamics and nonlinear observations of states. Although our applications did include nonlinear applications, our policies were still derived from linear approximations. It will be interesting to examine consequences of relaxing these assumptions. Although exact solutions will not be available, modern methods such as reinforcement learning could be applied to learn solutions to these extended control problems with computational costs. In none of these cases will families of optimal policies be analytically characterizable, as we did here, but our results can guide tests of how agents trade performance against frugal computation in complex tasks. We predict that the principles we discover here will generalize to these other situations.

Our framework could be extended in ways that benefit both artificial intelligence and neuroscience. Modern robots like NASA’s Valkyrie [46] and Boston Dynamics’ Atlas [47] demonstrate the level of performance modern hardware can support. However, bridging the gap to full autonomy demands breakthroughs in resource-efficient software. The slowdown of Moore’s Law heightens the urgency of this challenge, making it clear that ramping up computational resources on demand is no longer a sustainable way to transform multi-modal, high-dimensional, high-throughput data into representations that support decision-making. Control algorithms that embrace frugal inference offer a promising avenue for long-term scalability. This new paradigm challenges the traditional design of inference and control as independent modules, but this shift in perspective is well justified by how biological intelligence operates. Animals can turn noisy stimuli into actions that address a wide range of tasks using limited experience, relying on modest processing capacity, and consuming less energy than a light bulb [48]. Extensive theoretical and empirical evidence suggests that, over millions of years of evolution, the brain has developed mechanisms not only to encode noisy sensory inputs into resource-efficient representations [36, 38, 49–54], but also to flexibly re-code these representations through cognitive processes that are meta-regulated to match resource availability with environmental structure and task demands [25, 26, 55–59]. Although a complete quantitative theory of how sensorimotor intelligence arises in the brain remains elusive, ongoing efforts in this direction can inform the design of more capable, adaptive, and computationally-efficient machines. With appropriate modifications, the framework we developed to study information efficiency in uncertain environments may contribute in part to this broad and ambitious endeavor.

In future work we could specialize our frugal inference framework to include more biological detail, and thereby generate testable predictions for animal studies of sensory-motor control, where current explanations are largely limited to efficient sensory coding for perception, rather for control. For example, our loss function can be readily adapted to quantify the cost of inference in terms of the expected number of spikes a neural network needs for belief updating. This quantity has been characterized for spiking neural networks using probabilistic population codes in linear-Gaussian settings [60]. However, the brain evolved to solve tasks where the assumption of stationary, time-invariant transitions often breaks down. For example, foraging requires interpreting sensory cues whose reliability varies across space and time. Meta-learning [61] can help address this challenge by accelerating the adaptation of our frugal strategies to rapidly changing contexts. Enhanced with these modifications, our framework could inform theories of how neural systems integrate perception, inference, and control to produce action. It could also help explain how distinct cognitive strategies or personality types can be equally adaptive under a single set of conditions. Future work may also investigate how the complex properties that arise from jointly optimizing inference and control could be leveraged to improve the computational efficiency of model-based reinforcement learning. These methods have demonstrated improvements in data efficiency and generalization compared to model-free alternatives, but they typically require frequent re-planning, which can be computationally demanding [62]. We hypothesize that the free design subspace that emerges when a frugal agent leaves some epistemic uncertainty unresolved could be leveraged to accelerate online re-planning. For linear-Gaussian POMDPs, this subspace makes it easier to meet objectives that were not initially considered, while preserving performance on the original task. If this property extends to more complex dynamics, frugal versions of model-based reinforcement learning could substantially advance existing methods for handling control problems under dynamic, unforeseen constraints. Such challenges often arise in robot locomotion with complex actuators that enable flexible and adaptive contact with the environment [63, 64].

## 4 Methods

This section outlines the methodological foundations of our study. We begin by describing the modeling assumptions and derivations used to parameterize candidate strategies. Next, we present the numerical approach for identifying frugal strategies that solve our meta-cognitive POMDPs. Following this, we provide a mathematical analysis that reveals the free orthogonal transformation connecting members of the solution family. Finally, we detail the procedure for characterizing frugal strategies. Collectively, these methods enabled us to extract core principles that allow agents to balance performance and resource usage in POMDPs with linear-Gaussian dynamics.

### 4.1 Problem formulation

To investigate how the computational burden of inference influences rational behavior in partially observable environments, we propose a meta-cognitive variant of the POMDP framework. In this version, belief updating is no longer a fixed subroutine, but a cost-sensitive process optimized jointly with control (action selection). In general settings, the parameters defining the solution to this computationally constrained control problem can be complex, such as the weights of a recurrent neural network. However, these parameters are simpler and more interpretable for linear-Gaussian POMDPs, *i.e.* settings where the hidden state evolves according to stochastic linear dynamics, $s_{t}=Ds_{t-1}+Ea_{t-1}+w_{t-1}$, and observations are linear, noisy versions of the hidden state $o_{t}=s_{t}+v_{t}$. Here, the dynamics matrix D captures how unstable the state is, the input gain matrix E characterizes actuator responsiveness, $a_{t-1}$ is the action taken by the agent, $w_{t-1}$ is additive white Gaussian noise with isotropic covariance Q, and $v_{t}$ is additive white Gaussian noise with isotropic covariance R.

For meta-cognitive POMDPs with linear-Gaussian dynamics, finding the frugal strategy $Ж=\left\{\phi^{b},\phi^{a}\right\}$ that balances task performance, motion effort, and the information gained through inference boils down to solving the following optimization problem:

(2) $$\begin{array}{l} \;\underset{\left\{\phi^{b},\phi^{a}\right\}}{\min}\;E_{\tau ∼p(\tau )}\left[\sum_{t}s_{t}^{⊤}C_{s}s_{t}+a_{t}^{⊤}C_{a}a_{t}+C_{n}I\left(s_{t};b_{t}\right)\right]\\ \text{subject to}\;s_{t}=Ds_{t-1}+Ea_{t-1}+w_{t-1}\;;\;w_{t-1}∼N(0,Q)\\ \;o_{t}=s_{t}+v_{t}\;;\;v_{t}∼N(0,R)\\ \;b_{t}=f\left(o_{\leq t},a_{<t};\phi^{b}\right)\\ \;a_{t}=g\left(b_{t};\phi^{a}\right) \end{array}$$

where f defines the inference process that integrates new evidence under parameters $\phi^{b}$, and g specifies the control policy that maps the resulting beliefs to actions under parameters $\phi^{a}$. In the cost function, the penalties $\text{Ξ}=\left\{C_{s},C_{a},C_{n}\right\}$ determine the relative importance of the competing objectives: minimizing state deviations, reducing motion effort, and saving bits in the inference. Crucially, the expectation E is taken with respect to the probability distribution of trajectories $\tau =\left\{s_{0:T},o_{0:T},a_{0:T},b_{0:T}\right\}$ that the frugal strategy generates given the dynamics defined by the world properties Ω={D,E,Q,R}. We assume that both Ω and Ξ are known and change slowly. This enables the agent to decide how to compress previous evidence while observing the real consequences of its actions, and compensate for estimation errors that result from cheaper inference through additional motion effort.

### 4.2 Parameterizing the solution

Exact belief updating follows directly from recursive Bayesian inference:

$$b_{t}=p\left(s_{t}|o_{0},\cdots ,o_{t},a_{0},\cdots ,a_{t-1}\right)\propto p\left(o_{t}|s_{t}\right)\int p\left(s_{t}|s_{t-1},a_{t-1}\right)b_{t-1}\text{d}s_{t-1}$$

In this process, the belief is propagated through a transition model $p\left(s_{t}|s_{t-1},a_{t-1}\right)$ and updated using an observation model $p\left(o_{t}|s_{t}\right)$ via Bayes rule. In POMDPs with linear-Gaussian dynamics, this process is analytically tractable and yields a Gaussian posterior: $b_{t}=N\left({\hat{s}}_{t},P_{t}\right)$. The closed-form expressions for the posterior mean and covariance are:

(3) $${\hat{s}}_{t}=D{\hat{s}}_{t-1}+Ea_{t-1}+\left(DP_{t-1}D^{⊤}+Q\right){\left(DP_{t-1}D^{⊤}+Q+R\right)}^{-1}\left(o_{t}-\left(D{\hat{s}}_{t-1}+Ea_{t-1}\right)\right)$$

(4) $$P_{t}=\left(I-\left(DP_{t-1}D^{⊤}+Q\right){\left(DP_{t-1}D^{⊤}+Q+R\right)}^{-1}\right)\left(DP_{t-1}D^{⊤}+Q\right)$$

where $\tilde{\text{Ω}}=\{D,E,Q,R\}$ is the set of parameters describing how the agent assumes the hidden state evolves and generates observations. These closed-form expressions define the celebrated Kalman filter, and yield an exact posterior distribution when $\tilde{\text{Ω}}$ faithfully represents reality. Our agents capitalize on this analytical tractability but can tune the parameters $\tilde{\text{Ω}}$ to modulate inference quality.

Equations 3 and 4 admit further simplification. When the parameters $\tilde{\text{Ω}}$ are time-invariant, the posterior covariance converges to a steady-state value P. Upon convergence—and assuming actions are linear functions of the state estimate, $a_{t}=L{\hat{s}}_{t}$—Equation 3 takes the form of an exponential filter:

$${\hat{s}}_{t}=(D+EL){\hat{s}}_{t-1}+\left[\left(DPD^{⊤}+Q\right){\left(DPD^{⊤}+Q+R\right)}^{-1}\right]\left(o_{t}-(D+EL){\hat{s}}_{t-1}\right)\;\;=(D+EL){\hat{s}}_{t-1}+\beta \left(o_{t}-(D+EL){\hat{s}}_{t-1}\right)\;\;=[(D+EL)(I-\beta )]{\hat{s}}_{t-1}+\beta o_{t}\;\;=\text{Γ}{\hat{s}}_{t-1}+\beta o_{t}\;\;=\beta \sum_{i=0}^{t}{\text{Γ}}^{i}o_{t-i}$$

Therefore, for a meta-cognitive POMDP with linear-Gaussian dynamics, the parameters $\{\text{Γ},\beta \}=\phi^{b}$ completely define the inference process. Here, Γ indicates how much of the past should be remembered, while β scales observations to minimize estimation bias. We assume that actions are linear functions of the state estimate: $a_{t}=L{\hat{s}}_{t}$; thus, the control policy is fully parameterized by the control gain $\{L\}=\phi^{a}$. We refer to the parameters {Γ,β,L} that optimize problem 2 as the *frugal strategy*.

### 4.3 Computing frugal strategies

While we exploit the linear-Gaussian structure of the problem to compute and interpret frugal strategies, our approach differs from classic LQG control. In our meta-cognitive POMDP, the agent pays for every bit of information gained through inference, with a cost rate modulated by the parameter $C_{n}$ in the loss function. This incentivizes the agent to jointly optimize inference and control, which poses a challenge for the conventional LQG controller. When the belief fails to fully capture the history of past observations and actions, it cannot restore the Markov property that the past and the future are conditionally independent given the present. Since the LQG controller relies on this property to guarantee the optimality of its solutions, modulating inference quality undermines its effectiveness. Finding the frugal strategy that solves a meta-cognitive POMDP thus requires reasoning over a joint space of states and actions. To address this challenge, we create an augmented state variable $z_{t}={\left[s_{t},a_{t}\right]}^{⊤}$ that describes the joint evolution of states and actions:

$$z_{t}=\left[\begin{array}{cc} D & E\\ D\text{Ψ} & \text{Π}+E\text{Ψ} \end{array}\right]z_{t-1}+\left[\begin{array}{c} w_{t-1}\\ \text{Ψ}\left(w_{t-1}+v_{t}\right) \end{array}\right]=\text{M}z_{t-1}+\eta_{t-1};\eta_{t-1}\sim N(0,\Upsilon )$$

Here, M describes the base dynamics of the augmented state $z_{t}$, Υ characterizes the randomness in the joint space of states and actions, and $\left\{\text{Π}=L\text{Γ}L^{+},\text{Ψ}=L\beta \right\}$ represent the parameters of the controller’s input-output form: $a_{t}=\text{Π}a_{t-1}+\text{Ψ}o_{t}$. Here, the controller’s base dynamics Π defines how the actions evolve without new evidence, the observation sensitivity Ψ determines which dimensions can be attenuated or magnified without compromising eventual goal attainment, and the symbol ^+^ denotes the Moore–Penrose pseudoinverse. If $z_{t}$ can be stabilized by tuning the parameters {Π,Ψ}, its probability distribution reaches equilibrium and becomes $p\left(z_{t}\right)=N(0,\text{Σ})$ for all t. Therefore, at equilibrium, the entries of the steady-state covariance matrix $\text{Σ}=\left[\begin{array}{cc} {\text{Σ}}_{s} & {\text{Σ}}_{as}\\ {\text{Σ}}_{sa} & {\text{Σ}}_{a} \end{array}\right]$ fully define the components of the loss function. As a result, at equilibrium, our computationally constrained control problem becomes:

(5) $$\underset{\left\{\text{Π},\text{Ψ}\right\}}{\min}\left\{\mathrm{Tr}\left(C_{s}{\text{Σ}}_{s}+C_{a}{\text{Σ}}_{a}\right)+C_{n}\frac{1}{2}\log_{2}\frac{\det\;\left({\text{Σ}}_{s}\right)\det\;\left({\text{Σ}}_{a}\right)}{\det\;(\text{Σ})}\right\}$$

We solve problem 5 using stochastic gradient descent, which iteratively adjusts the parameters {Π,Ψ} to minimize the loss. We verify that the candidate solutions produce a positive definite covariance matrix Σ and a transition matrix M with stable eigenvalues throughout the optimization process. These conditions guarantee that the trajectory distribution of states and actions is well-defined and that the system can reach the target state. Additionally, we monitor the Hessian of the objective function to ensure that the solutions are locally optimal, stable, and meaningful. The numerical optimization method described here produces the landscapes shown in Figures 2B and 2C of the main text. While those results correspond to a scalar task, the approach generalizes to multivariate problems, as demonstrated by the illustrative tasks in Section 2.4.

### 4.4 Recovering the complete solution family

The frugal strategy $\left\{{\text{Π}}^{*},{\text{Ψ}}^{*}\right\}$ that solves problem 5 induces a unique covariance matrix ${\text{Σ}}^{*}$. However, the reverse is not true: a given matrix ${\text{Σ}}^{*}$ may correspond to multiple combinations of ${\text{Π}}^{*}$ and ${\text{Ψ}}^{*}$. To understand the conditions under which this occurs, we analyze the structure of the discrete-time Lyapunov equation that ${\text{Σ}}^{*}$ satisfies:

(6) $$\left[\begin{array}{cc} {\text{Σ}}_{s}^{*} & {\text{Σ}}_{as}^{*}\\ {\text{Σ}}_{sa}^{*} & {\text{Σ}}_{a}^{*} \end{array}\right]=\left[\begin{array}{cc} D & E\\ D{\text{Ψ}}^{*} & {\text{Π}}^{*}+E{\text{Ψ}}^{*} \end{array}\right]\left[\begin{array}{cc} {\text{Σ}}_{s}^{*} & {\text{Σ}}_{as}^{*}\\ {\text{Σ}}_{sa}^{*} & {\text{Σ}}_{a}^{*} \end{array}\right]{\left[\begin{array}{cc} D & E\\ D{\text{Ψ}}^{*} & {\text{Π}}^{*}+E{\text{Ψ}}^{*} \end{array}\right]}^{⊤}+\left[\begin{array}{cc} Q & Q{\text{Ψ}}^{*⊤}\\ {\text{Ψ}}^{*}Q & {\text{Ψ}}^{*}(Q+R){\text{Ψ}}^{*⊤} \end{array}\right]$$

Solving equation 6 element-wise shows that the frugal strategy $\left\{{\text{Π}}^{*},{\text{Ψ}}^{*}\right\}$ satisfies a generalized ellipsoidal constraint:

(7) $${\text{Σ}}_{a}^{*}={\text{Π}}^{*}{\text{Σ}}_{a}^{*}{\text{Π}}^{*⊤}+{\text{Ψ}}^{*}{\text{Σ}}_{sa}^{*⊤}+{\text{Σ}}_{sa}^{*}{\text{Ψ}}^{*⊤}+{\text{Ψ}}^{*}\left(R-{\text{Σ}}_{s}^{*}\right){\text{Ψ}}^{*⊤}$$

with $R\geq {\text{Σ}}_{s}^{*}$ and ${\text{Ψ}}^{*}=\left({\text{Σ}}_{sa}^{*}-{\text{Π}}^{*}\left({\text{Σ}}_{sa}^{*}D^{⊤}+{\text{Σ}}_{a}^{*}E^{⊤}\right)\right){\text{Σ}}_{s}^{*-1}$.

Consequently, finding ${\text{Π}}^{*}$ as a function of ${\text{Σ}}^{*}$ and ${\text{Ψ}}^{*}$ requires solving a quadratic form:

$${\text{Π}}^{*}F_{2}{\text{Π}}^{*⊤}+\text{Π}F_{1}+F_{1}^{⊤}{\text{Π}}^{*⊤}=F_{0}$$

here, $F_{0}$, $F_{1}$, and $F_{2}$ are functions of ${\text{Σ}}^{*}$ and the world properties {D,E,Q,R}. This quadratic form can be rearranged as:

(8) $$\left({\text{Π}}^{*}+F_{1}^{⊤}F_{2}^{-1}\right)F_{2}{\left({\text{Π}}^{*}+F_{1}^{⊤}F_{2}^{-1}\right)}^{⊤}=F_{0}+F_{1}^{⊤}F_{2}^{-⊤}F_{1}$$

The right side of equation 8 quantifies how much epistemic uncertainty remains after updating the belief $b_{t}=N\left({\hat{s}}_{t},P\right)$ with the most recent observation $o_{t}$. That is:

(9) $$\begin{array}{l} F_{0}+F_{1}^{⊤}F_{2}^{-⊤}F_{1}\propto \text{Cov}\left(s_{t}-\langle s_{t}|{\hat{s}}_{t}\rangle \right)\text{Cov}{\left(\langle s_{t}|{\hat{s}}_{t}\rangle \right)}^{-1}\\ \;-\text{Cov}\left(s_{t+1}-\langle s_{t+1}|{\hat{s}}_{t},o_{t+1}\rangle \right)\text{Cov}{\left(\langle s_{t+1}|{\hat{s}}_{t},o_{t+1}\rangle \right)}^{-1} \end{array}$$

with 〈⋅〉 denoting the expectation taken with respect to the probability distribution of trajectories that the frugal strategy generates given the dynamics defined by the true world properties Ω={D,E,Q,R}. Equation 9 quantifies the difference between the uncertainty that remains unexplained about $s_{t}$ given its estimate ${\hat{s}}_{t}=\int s_{t}b\left(s_{t}\right)\text{d}s_{t}$ and the uncertainty that remains unexplained about the next state given the current estimate and the next observation. When this difference is zero, equation 8 takes a linear form and the solution is unique: ${\text{Π}}^{*}=-F_{1}^{⊤}F_{2}^{-1}$. However, when the agent pays for every bit of information gained through inference, the cost of mitigating epistemic uncertainty may outweigh its benefits in solving the task. When this happens, the unresolved uncertainty yields slack in the ellipsoidal constraint (Equation 7) that a frugal strategy must satisfy to balance state, action, and inference costs. This additional freedom gives rise to multiple combinations of lossy inference and error-aware control that can solve the planning problem equally well.

To recover the complete solution family that emerges when epistemic uncertainty remains unresolved, we calculate the eigenvalue decomposition of $F_{2}$ on the left side of equation 8 and the eigenvalue decomposition of $\xi =F_{0}+F_{1}^{⊤}F_{2}^{-⊤}F_{1}$ on the right side:

$$\left({\text{Π}}^{*}+F_{1}^{⊤}F_{2}^{-1}\right)U_{F_{2}}{\text{Λ}}_{F_{2}}^{\frac{1}{2}}{\text{Λ}}_{F_{2}}^{\frac{1}{2}}U_{F_{2}}^{⊤}{\left({\text{Π}}^{*}+F_{1}^{⊤}F_{2}^{-1}\right)}^{⊤}=U_{\xi }{\text{Λ}}_{\xi }^{\frac{1}{2}}{\text{Λ}}_{\xi }^{\frac{1}{2}}U_{\xi }^{⊤}$$

This decomposition reveals a free orthogonal transformation Θ on the joint space of states and actions:

$$\left({\text{Π}}^{*}+F_{1}^{⊤}F_{2}^{-1}\right)U_{F_{2}}{\text{Λ}}_{F_{2}}^{\frac{1}{2}}{\text{Λ}}_{F_{2}}^{\frac{1}{2}}U_{F_{2}}^{⊤}{\left({\text{Π}}^{*}+F_{1}^{⊤}F_{2}^{-1}\right)}^{⊤}=U_{\xi }{\text{Λ}}_{\xi }^{\frac{1}{2}}{\text{ΘΘ}}^{⊤}{\text{Λ}}_{\xi }^{\frac{1}{2}}U_{\xi }^{⊤}$$

We can use this transformation to parameterize the frugal strategies in the solution family:

$$\left({\text{Π}}^{*}+F_{1}^{⊤}F_{2}^{-1}\right)U_{F_{2}}{\text{Λ}}_{F_{2}}^{\frac{1}{2}}=U_{\xi }{\text{Λ}}_{\xi }^{\frac{1}{2}}\text{Θ}\;\to \;{\text{Π}}_{\text{Θ}}^{*}=U_{\xi }{\text{Λ}}_{\xi }^{\frac{1}{2}}\text{Θ}{\text{Λ}}_{F_{2}}^{-\frac{1}{2}}U_{F_{2}}^{-1}-F_{1}^{⊤}F_{2}^{-1}\;\;{\text{Ψ}}_{\text{Θ}}^{*}=\left({\text{Σ}}_{sa}-{\text{Π}}_{\text{Θ}}^{*}\left({\text{Σ}}_{sa}D^{⊤}+{\text{Σ}}_{a}E^{⊤}\right)\right){\text{Σ}}_{s}^{-1}$$

By construction, each family member $\left\{{\text{Π}}_{\text{Θ}}^{*},{\text{Ψ}}_{\text{Θ}}^{*}\right\}$ satisfies the ellipsoidal constraint (Equation 7) and, thus, results in the same bounded optimal covariance matrix ${\text{Σ}}^{*}$ whose entries fully define the loss function in problem 5. This explains why all family members perform equally well given the properties $\left\{D,E,Q,R,C_{s},C_{a},C_{n}\right\}$ for which they are optimized. However, due to differences in their temporal structure, the solutions vary in how the agent models the world to integrate new evidence, offsets estimation errors, and generalizes to novel settings. For high-level implications, see Section 2.3.2, where we outline intuition and applications of this mathematical analysis.

### 4.5 Interpreting frugal strategies

The first step to interpret a frugal strategy $\left\{\text{Π}=L\text{Γ}L^{+},\text{Ψ}=L\beta \right\}$ is to recover the parameters of the inference. We recover the filter’s memory factor using $\text{Γ}=\left(\beta {\text{Ψ}}^{+}\right)\text{Π}\left(\text{Ψ}\beta^{+}\right)$. The observation scaling factor β is a degenerate parameter because its effects can be neutralized by the control gain L. To address this degeneracy, we let β be the value that minimizes estimation bias. The next step is to identify the generative model that shapes the belief $b_{t}=N\left({\hat{s}}_{t},P;\text{Γ},\beta \right)$. Our agents engage in recursive Bayesian inference but distort the generative model to modulate inference quality; thus, their subjective posterior mean and covariance have the following closed-form expressions:

(10) $${\hat{s}}_{t}=(D+EL){\hat{s}}_{t-1}+\beta \left(o_{t}-(D+EL){\hat{s}}_{t-1}\right)\;\;=(D+EL)(I-\beta ){\hat{s}}_{t-1}+\beta o_{t}\;\;=\text{Γ}\;{\hat{s}}_{t-1}+\beta o_{t}$$

(11) $$P=\left(I-\left(DPD^{⊤}+Q\right){\left(DPD^{⊤}+Q+R\right)}^{-1}\right)\left(DPD^{⊤}+Q\right)$$

If the agent’s assumptions $\tilde{\text{Ω}}=\{D,E,Q,R\}$ truly reflected reality, the posterior covariance P would equal the mean squared estimation error:

(12) $$P={\text{Σ}}_{e}=E_{\tau \sim p(\tau )}\left[{\left(s_{t}-{\hat{s}}_{t}\right)}^{2}|o_{\leq t},a_{<t}\right]\;\;={\text{Σ}}_{s}-{\text{Σ}}_{s\hat{s}}-{\text{Σ}}_{s\hat{s}}^{⊤}+{\text{Σ}}_{\hat{s}}\;\;={\text{Σ}}_{s}-\left(\beta {\text{Ψ}}^{+}\right){\text{Σ}}_{sa}-{\text{Σ}}_{sa}^{⊤}{\left(\beta {\text{Ψ}}^{+}\right)}^{⊤}+\left(\beta {\text{Ψ}}^{+}\right){\text{Σ}}_{a}{\left(\beta {\text{Ψ}}^{+}\right)}^{⊤}$$

with the expectation E taken with respect to the probability distribution of trajectories $\tau =\left\{s_{0:T},o_{0:T},a_{0:T},b_{0:T}\right\}$ that obey the dynamics defined by the true world properties Ω={D,E,Q,R}.

We use equations 10, 11, and 12 to recover $\tilde{\text{Ω}}$. To this end, we treat actuator responsiveness and sensor noise covariance as intrinsic properties of the agent; accordingly, in the assumed world model $\tilde{\text{Ω}}$ they are set equal to their true values, E=E and R=R. We then derive D and Q as follows:

$$D={\left(I-\beta \right)}^{-1}\text{Γ}-E\;L\;Q={\left(R^{-1}\left(R^{-1}-{\text{Σ}}_{e}\right)R^{-1}\right)}^{-1}-D{\text{Σ}}_{e}D^{⊤}-R$$

This derivation allowed us to thoroughly characterize the solutions to problem 5, leading to the results presented in Figures 3C and 4 of the main text.

## Code availability.

All code developed for this work is publicly available at https://github.com/ItzelCoral/Frugal_Rationality.

## References

[1] Lauri M., Hsu D., Pajarinen J.: Partially observable markov decision processes in robotics: A survey. IEEE Transactions on Robotics 39(1), 21–40 (2022)

[2] Kurniawati H.: Partially observable markov decision processes and robotics. Annual Review of Control, Robotics, and Autonomous Systems 5(1), 253–277 (2022)

[3] Ross S., Pineau J., Chaib-draa B., Kreitmann P.: A bayesian approach for learning and planning in partially observable markov decision processes. Journal of Machine Learning Research 12(5) (2011)

[4] Lim M.H., Becker T.J., Kochenderfer M.J., Tomlin C.J., Sunberg Z.N.: Optimality guarantees for particle belief approximation of pomdps. Journal of Artificial Intelligence Research 77, 1591–1636 (2023)

[5] Watter M., Springenberg J., Boedecker J., Riedmiller M.: Embed to control: A locally linear latent dynamics model for control from raw images. Advances in neural information processing systems 28 (2015)

[6] Ha D., Schmidhuber J.: World models. arXiv preprint arXiv:1803.10122 (2018)

[7] Igl M., Zintgraf L., Le T.A., Wood F., Whiteson S.: Deep variational reinforcement learning for pomdps. In: International Conference on Machine Learning, pp. 2117–2126 (2018). PMLR

[8] Hafner D., Lillicrap T., Fischer I., Villegas R., Ha D., Lee H., Davidson J.: Learning latent dynamics for planning from pixels. In: International Conference on Machine Learning, pp. 2555–2565 (2019). PMLR

[9] Hafner D., Pasukonis J., Ba J., Lillicrap T.: Mastering diverse control tasks through world models. Nature, 1–7 (2025) 10.1038/s41586-025-08744-2 PMC12003158 40175544

[10] Simon H.A.: A behavioral model of rational choice. The quarterly journal of economics, 99–118 (1955)

[11] Russell S.J., Subramanian D.: Provably bounded-optimal agents. Journal of Artificial Intelligence Research 2, 575–609 (1994)

[12] Russell S., Wefald E.: Principles of metareasoning. Artificial intelligence 49(1–3), 361–395 (1991)

[13] Cox M.T.: Metacognition in computation: A selected research review. Artificial intelligence 169(2), 104–141 (2005)

[14] Horvitz E.J.: Reasoning about beliefs and actions under computational resource constraints. arXiv preprint arXiv:1304.2759 (2013)

[15] Tishby N., Polani D.: Information theory of decisions and actions. In: Perception-action Cycle: Models, Architectures, and Hardware, pp. 601–636. Springer, New York, NY (2010)

[16] Rubin J., Shamir O., Tishby N.: Trading value and information in mdps. In: Decision Making with Imperfect Decision Makers, pp. 57–74. Springer, Berlin, Heidelberg (2012)

[17] Ortega P.A., Braun D.A.: Thermodynamics as a theory of decision-making with information-processing costs. Proceedings of the Royal Society A: Mathematical, Physical and Engineering Sciences 469(2153), 20120683 (2013)

[18] Ortega P.A., Braun D.A., Dyer J., Kim K.-E., Tishby N.: Information-theoretic bounded rationality. arXiv preprint arXiv:1512.06789 (2015)

[19] Tsiotras P.: Bounded rationality in learning, perception, decision-making, and stochastic games. In: Handbook of Reinforcement Learning and Control, pp. 491–523. Springer, Germany (2021)

[20] Grau-Moya J., Leibfried F., Genewein T., Braun D.A.: Planning with information-processing constraints and model uncertainty in markov decision processes. In: Machine Learning and Knowledge Discovery in Databases: European Conference, ECML PKDD 2016, Riva del Garda, Italy, September 19–23, 2016, Proceedings, Part II 16, pp. 475–491 (2016). Springer

[21] Binz M., Gershman S.J., Schulz E., Endres D.: Heuristics from bounded metalearned inference. Psychological review 129(5), 1042 (2022) 34990160 10.1037/rev0000330

[22] Lancia G.L., Eluchans M., D’Alessandro M., Spiers H.J., Pezzulo G.: Humans account for cognitive costs when finding shortcuts: An information-theoretic analysis of navigation. PLOS Computational Biology 19(1), 1010829 (2023) 10.1371/journal.pcbi.1010829 PMC9851521 36608145

[23] Moskovitz T., Miller K., Sahani M., Botvinick M.M.: A unified theory of dual-process control. arXiv preprint arXiv:2211.07036 (2022)

[24] Kool W., Gershman S.J., Cushman F.A.: Planning complexity registers as a cost in metacontrol. Journal of cognitive neuroscience 30(10), 1391–1404 (2018) 29668390 10.1162/jocn_a_01263

[25] Ho M.K., Abel D., Correa C.G., Littman M.L., Cohen J.D., Griffiths T.L.: People construct simplified mental representations to plan. Nature 606(7912), 129–136 (2022) 35589843 10.1038/s41586-022-04743-9

[26] Ongchoco J.D.K., Knobe J., Jara-Ettinger J.: People’s thinking plans adapt to the problem they’re trying to solve. Cognition 243, 105669 (2024) 38039797 10.1016/j.cognition.2023.105669

[27] Pedram A.R., Stefarr J., Funada R., Tanaka T.: Rationally inattentive path-planning via rrt. In: 2021 American Control Conference (ACC), pp. 3440–3446 (2021). IEEE

[28] Mazzaglia P., Verbelen T., Dhoedt B.: Contrastive active inference. Advances in neural information processing systems 34, 13870–13882 (2021)

[29] Pacelli V., Majumdar A.: Robust control under uncertainty via bounded rationality and differential privacy. In: 2022 International Conference on Robotics and Automation (ICRA), pp. 3467–3474 (2022). IEEE

[30] Howard R.A.: Information value theory. IEEE Transactions on systems science and cybernetics 2(1), 22–26 (2007)

[31] Gershman S., Wilson R.: The neural costs of optimal control. Advances in neural information processing systems 23 (2010)

[32] Genewein T., Leibfried F., Grau-Moya J., Braun D.A.: Bounded rationality, abstraction, and hierarchical decision-making: An information-theoretic optimality principle. Frontiers in Robotics and AI 2, 27 (2015)

[33] Schmid G., Gottwald S., Braun D.A.: Bounded rational decision networks with belief propagation. Neural Computation 37(1), 76–127 (2024) 39383021 10.1162/neco_a_01719

[34] Sims C.A.: Implications of rational inattention. Journal of monetary Economics 50(3), 665–690 (2003)

[35] Tatikonda S., Mitter S.: Control under communication constraints. IEEE Transactions on automatic control 49(7), 1056–1068 (2004)

[36] Susemihl A.K., Meir R., Opper M.: Optimal neural codes for control and estimation. Advances in neural information processing systems 27 (2014)

[37] Gershman S.J., Horvitz E.J., Tenenbaum J.B.: Computational rationality: A converging paradigm for intelligence in brains, minds, and machines. Science 349(6245), 273–278 (2015) 26185246 10.1126/science.aac6076

[38] Grujic N., Brus J., Burdakov D., Polania R.: Rational inattention in mice. Science advances 8(9), 8935 (2022) 10.1126/sciadv.abj8935 PMC8896787 35245128

[39] Bertsekas D.: Dynamic Programming and Optimal Control: Volume I vol. 4. Athena scientific, United States (2012)

[40] Tang Y., Zheng Y., Li N.: Analysis of the optimization landscape of linear quadratic gaussian (lqg) control. Mathematical Programming 202(1), 399–444 (2023)

[41] Hu B., Zhang K., Li N., Mesbahi M., Fazel M., Başar T.: Toward a theoretical foundation of policy optimization for learning control policies. Annual Review of Control, Robotics, and Autonomous Systems 6(1), 123–158 (2023)

[42] Todorov E.: Stochastic optimal control and estimation methods adapted to the noise characteristics of the sensorimotor system. Neural computation 17(5), 1084–1108 (2005) 15829101 10.1162/0899766053491887 PMC1550971

[43] Boominathan L., Pitkow X.: Phase transitions in when feedback is useful. Advances in Neural Information Processing Systems 35, 10849–10861 (2022)

[44] Brunskill E., Kaelbling L.P., Lozano-Perez T., Roy N.: Continuous-state pomdps with hybrid dynamics. In: ISAIM (2008)

[45] Todorov E.: Linearly-solvable markov decision problems. Advances in neural information processing systems 19 (2006)

[46] Radford N.A., Strawser P., Hambuchen K., Mehling J.S., Verdeyen W.K., Donnan A.S., Holley J., Sanchez J., Nguyen V., Bridgwater L., : Valkyrie: Nasa’s first bipedal humanoid robot. Journal of Field Robotics 32(3), 397–419 (2015)

[47] Guizzo E.: By leaps and bounds: An exclusive look at how boston dynamics is redefining robot agility. IEEE Spectrum 56(12), 34–39 (2019)

[48] Padamsey Z., Katsanevaki D., Dupuy N., Rochefort N.L.: Neocortex saves energy by reducing coding precision during food scarcity. Neuron 110(2), 280–296 (2022) 34741806 10.1016/j.neuron.2021.10.024 PMC8788933

[49] Attneave F.: Some informational aspects of visual perception. Psychological review 61(3), 183 (1954) 13167245 10.1037/h0054663

[50] Barlow H.B., : Possible principles underlying the transformation of sensory messages. Sensory communication 1(01), 217–233 (1961)

[51] Pitkow X., Meister M.: Decorrelation and efficient coding by retinal ganglion cells. Nature neuroscience 15(4), 628–635 (2012) 22406548 10.1038/nn.3064 PMC3725273

[52] Wei X.-X., Stocker A.A.: A bayesian observer model constrained by efficient coding can explain’anti-bayesian’percepts. Nature neuroscience 18(10), 1509–1517 (2015) 26343249 10.1038/nn.4105

[53] Park I.M., Pillow J.W.: Bayesian efficient coding. BioRxiv, 178418 (2017)

[54] Zheng J., Meister M.: The unbearable slowness of being: Why do we live at 10 bits/s? Neuron 113(2), 192–204 (2025) 39694032 10.1016/j.neuron.2024.11.008 PMC11758279

[55] Laughlin S.: A simple coding procedure enhances a neuron’s information capacity. Zeitschrift für Naturforschung c 36(9–10), 910–912 (1981) 7303823

[56] Tavoni G., Doi T., Pizzica C., Balasubramanian V., Gold J.: The complexity dividend: when sophisticated inference matters. BioRxiv, 563346 (2019)

[57] Vul E., Goodman N., Griffiths T.L., Tenenbaum J.B.: One and done? optimal decisions from very few samples. Cognitive science 38(4), 599–637 (2014) 24467492 10.1111/cogs.12101

[58] Salmasi M., Sahani M.: Learning neural codes for perceptual uncertainty. In: 2022 IEEE International Symposium on Information Theory (ISIT), pp. 2463–2468 (2022). IEEE

[59] Lan D.C., Hunt L.T., Summerfield C.: Goal-directed navigation in humans and deep reinforcement learning agents relies on an adaptive mix of vector-based and transition-based strategies. Plos Biology 23(7), 3003296 (2025) 10.1371/journal.pbio.3003296 PMC12324678 40729396

[60] Beck J.M., Latham P.E., Pouget A.: Marginalization in neural circuits with divisive normalization. Journal of Neuroscience 31(43), 15310–15319 (2011) 22031877 10.1523/JNEUROSCI.1706-11.2011 PMC3230133

[61] Finn C., Abbeel P., Levine S.: Model-agnostic meta-learning for fast adaptation of deep networks. In: International Conference on Machine Learning, pp. 1126–1135 (2017). PMLR

[62] Nagabandi A., Konolige K., Levine S., Kumar V.: Deep dynamics models for learning dexterous manipulation. In: Conference on Robot Learning, pp. 1101–1112 (2020). PMLR

[63] Hwangbo J., Lee J., Dosovitskiy A., Bellicoso D., Tsounis V., Koltun V., Hutter M.: Learning agile and dynamic motor skills for legged robots. Science Robotics 4(26), 5872 (2019) 10.1126/scirobotics.aau5872 33137755

[64] Hoeller D., Rudin N., Sako D., Hutter M.: Anymal parkour: Learning agile navigation for quadrupedal robots. Science Robotics 9(88), 7566 (2024) 10.1126/scirobotics.adi7566 38478592

